# A Novel Framework to Address the Complexities of Housing Insecurity and Its Associated Health Outcomes and Inequities: “Give, Partner, Invest”

**DOI:** 10.3390/ijerph20146349

**Published:** 2023-07-12

**Authors:** Sonika Bhatnagar, John Lovelace, Ray Prushnok, Justin Kanter, Joan Eichner, Dan LaVallee, James Schuster

**Affiliations:** 1 UPMC Insurance Services Division, 600 Grant Street, Pittsburgh, PA 15219, USA; 2 Department of Pediatrics, University of Pittsburgh School of Medicine, UPMC Children’s Hospital of Pittsburgh, 4401 Penn Avenue, Pittsburgh, PA 15224, USA; 3 UPMC Center for Social Impact, 600 Grant Street, 40th Floor, Pittsburgh, PA 15219, USA; 4 UPMC Center for High-Value Health Care, 600 Grant Street, 40th Floor, Pittsburgh, PA 15219, USA

**Keywords:** housing insecurity, affordable housing, health outcomes, health inequities, health-related social needs, healthcare systems, community-based organizations

## Abstract

The association between housing insecurity and reduced access to healthcare, diminished mental and physical health, and increased mortality is well-known. This association, along with structural racism, social inequities, and lack of economic opportunities, continues to widen the gap in health outcomes and other disparities between those in higher and lower socio-economic strata in the United States and throughout the advanced economies of the world. System-wide infrastructure failures at municipal, state, and federal government levels have inadequately addressed the difficulty with housing affordability and stability and its associated impact on health outcomes and inequities. Healthcare systems are uniquely poised to help fill this gap and engage with proposed solutions. Strategies that incorporate multiple investment pathways and emphasize community-based partnerships and innovation have the potential for broad public health impacts. In this manuscript, we describe a novel framework, “Give, Partner, Invest,” which was created and utilized by the University of Pittsburgh Medical Center (UPMC) Insurance Services Division (ISD) as part of the Integrated Delivery and Finance System to demonstrate the financial, policy, partnership, and workforce levers that could make substantive investments in affordable housing and community-based interventions to improve the health and well-being of our communities. Further, we address housing policy limitations and infrastructure challenges and offer potential solutions.

## 1. Introduction

According to the National Homelessness Law Center (formerly the National Law Center on Homelessness & Poverty (NLCHP), the leading cause of homelessness is an absence of affordable housing and housing insecurity [1]. In the United States, 37 million households live in housing that is considered “unaffordable” for their income, and 3.6 million households face eviction summonses annually [2]. With the end of the COVID-19 Public Health Emergency and Federal Eviction Moratorium, COVID-19-induced economic stressors, inflation [3,4], and the tightening of the housing and rental markets, means that even more households are at risk of housing displacement and experiencing homelessness [5]. According to the U.S. Department of Housing and Urban Development (HUD) 2022 Annual Homeless Assessment Report to Congress, there continues to be an overrepresentation of people experiencing housing insecurity who identify as Black, African American, or African, Native American, and Pacific Islander [5]. The association between housing insecurity and reduced access to healthcare, diminished mental and physical health, and increased mortality is well-known [6]. This association, along with structural racism, social inequities, and a lack of economic opportunities, continues to widen the gap between health outcomes and inequities.

While addressing housing insecurity has become a priority for both public health and healthcare providers, most housing efforts have been in the form of secondary or tertiary rather than primary prevention. By definition, secondary prevention focuses on preventing the worsening of the existing housing crisis, and tertiary focuses on avoiding further complications by providing housing for those chronically experiencing housing insecurities. On the other hand, primary prevention focuses on improving housing affordability and stability to avert housing insecurity and displacement [6]. As of the November 2022 Systematic Review on “The Association of Promoting Housing Affordability and Stability with Improved Health Outcomes”, there has been a lack of relevant studies on the association between health outcomes and health-system-partnered, structured primary prevention [1]. In advanced economies of the world, public–private partnerships have been an important mechanism for improving housing conditions and leading primary prevention efforts for housing affordability and stability [7]. For example, in Australia, Ireland, and the United Kingdom, successful affordable housing programs are financed, developed, and operated in partnership with private sector organizations [7]. In the United States, historically, public funding for housing development, preservation, and assistance has been inadequate to address the growing affordable housing demands [8,9]. Given current system-wide infrastructure failures to address difficulties with housing affordability and stability and its associated impact on health outcomes and inequities, healthcare systems in the United States are increasingly seen as key private sector collaborators and financers [10]. 

In fact, healthcare systems are well-positioned to support and lead primary prevention efforts to address housing shortages, improve housing conditions, and develop public health infrastructure that is necessary to coordinate, evaluate, and sustain these interventions [11]. Healthcare systems already manage healthcare insurance for large and diverse populations and broadly screen for health-related social needs (HRSNs). These systems have robust care management workforces to address identified unmet housing needs and aid in providing services, resources, and support to social and community programs. Further, to an extent, healthcare systems can choose to utilize healthcare dollars to supplement funding for housing initiatives and redesign payment incentives to address health-related social needs. Healthcare systems affiliated with medical facilities and universities, such as those that operate as an integrated delivery and finance system (IDFS), have an opportunity to serve as an anchor for economic stimulus and community investment to address housing insecurities and subsequently improve health outcomes and reduce health inequities [12]. An IDFS is an integrated healthcare system where clinical providers and health insurance providers work together to improve clinical outcomes and optimize their clinical and financial performance.

Over time, healthcare systems in the United States have allocated more resources to primary housing interventions [10] as the association between housing and health outcomes has become evident [13]. Healthcare system housing-related investments coincide with federal and state reforms that aim to rebalance spending between healthcare and social services and improve equitable long-term health outcomes [14]. While these investments have increased, healthcare systems are still learning the nuances of navigating outdated or evolving regulations, financing housing initiatives, and building impactful community partnerships and well-integrated social and healthcare service networks. Moving forward, healthcare systems need to understand how to optimize these workforce levers within finance, policy, and partnerships to implement cohesive and impactful housing strategies that can positively impact public health and inequities. 

In this commentary, we describe a novel framework to improve housing affordability and stability and avert displacement and homelessness and its associated health outcomes and inequities, “Give, Partner, Invest.” We describe the etiology behind the development of this framework by the healthcare system, UPMC Insurance Services Division (ISD), headquartered in Pittsburgh, Pennsylvania, and part of the UPMC Integrated Delivery and Finance System affiliated with UPMC and the University of Pittsburgh Schools of the Health Sciences. We describe how UPMC ISD currently utilizes this framework to address the primary prevention of housing insecurity and the associated social and health impacts of these efforts. We also highlight the policy and infrastructure challenges we have faced thus far and offer potential solutions. While our framework and experiences are based upon the United States healthcare system and are local to our community, we propose this framework as a tool for healthcare systems in advanced economies of the world, including government, public health, and community stakeholders in their efforts to address unmet housing needs and their associated impact on public health and inequities. 

## 2. Rationale for Framework Creation, “Give, Partner, Invest”

In 2010, UPMC ISD partnered with the local Department of Housing and Urban Development (HUD) to create one of its first housing programs for those with public insurance, Medicaid, and experiencing homelessness, called Cultivating Health for Success (CHFS). HUD was created as part of President Lyndon B. Johnson’s War on Poverty and was established in 1965 as a federal agency to be responsible for policy and programs to address America’s housing needs, improve and develop the nation’s communities, and enforce fair housing laws [15]. Medicaid is a public insurance program that is federally funded but administered by each state and provides healthcare to low-income families and individuals, including children, parents, pregnant women, seniors, and people with disabilities. The CHFS program combines permanent supportive housing, a Housing First approach [16], with intensive, highly coordinated healthcare services to focus on the primary prevention of housing insecurity and improve health outcomes and inequities (Housing First is an assistance approach that prioritizes providing permanent housing to people experiencing homelessness). A five-year internal review found that individuals who participated in the CHFS program demonstrated an increase in their utilization of preventive and necessary medical care and a decrease in unplanned healthcare services and costs. These results, in part, fostered the continued expansion of UPMC ISD’s housing investments. 

Simultaneously, changes occurred within the state of Pennsylvania’s Medicaid program that contributed to accelerating these housing investments. In 2015, Pennsylvania opted into the federal Medicaid expansion, and this allowed for an additional 1.7 million people to qualify for Medicaid insurance [17]. Soon after, Pennsylvania began to require Medicaid Managed Care Organizations (MCOs) or health plans, such as the Medicaid insurance at UPMC ISD, to enter into value-based payment arrangements with contracted healthcare providers [18]. (Value-based healthcare is a healthcare delivery model in which providers, including hospitals and physicians, are paid based on patient outcomes.) MCOs would later be required to incorporate community-based organization (CBO) partnerships within these arrangements [18]. In 2018, to rebalance spending between nursing facility care and home and community-based services, the state of Pennsylvania launched Community HealthChoices (CHC): an MCO for adults with Medicaid insurance who required long-term services and support or were dually eligible for Medicaid and Medicare. (Medicare is federal health insurance for people who are 65 or older, certain younger people with disabilities, or people with end-stage renal disease) [19]. As a result, UPMC ISD served as two of Pennsylvania’s largest Medicaid MCOs for behavioral and physical health insurance and one of three MCOs in Pennsylvania to provide CHC insurance. This Medicaid transformation resulted in a significant increase in the number of people with UPMC Medicaid insurance, many of whom were experiencing difficulties with housing affordability and stability [20]. For example, in 2017, Pennsylvania had an estimated shortage of 98,000 housing units [21]—including a shortage of 27,000 housing units in the city of Pittsburgh [22]—and rapidly rising rent and eviction rates [23].

To address these unmet housing needs, its associated impact on health outcomes and inequities, and to align a new portfolio of Housing First and community interventions across UPMC’s charitable mission, healthcare system operations, and financial investments, UPMC ISD created the framework “Give, Partner, Invest”. To lead the adoption of this framework and continually evaluate new programs and innovations into their impact on health outcomes and inequities, the UPMC Center for Social Impact was created from a cross-disciplinary team of experts in policy, evaluation, and clinical health. This Center also served as a liaison to the government, community partners, residents of the community, and community service providers to ensure the success of its community-based initiatives.

## 3. “Give”

Healthcare systems cannot use healthcare premiums from Medicaid or Medicare programs to directly pay for housing (although policy waivers approved in some states have created new opportunities to use Medicaid funding to directly finance housing) [24]. However, alternative financial mechanisms are available. In the United States, non-profit healthcare systems maintain a tax-exempt status with the Internal Revenue Service, in part, by conducting routine community assessments and addressing identified health-related social needs. As a result, UPMC ISD has prioritized charitable contributions for affordable housing developments and additional programs to strengthen community-based safety net support through UPMC’s community benefits initiative. For example, UPMC ISD funded housing developments with contributions to state-run initiatives such as Pennsylvania’s Neighborhood Assistance Program [25]. Through Pennsylvania’s Neighborhood Assistance Program, private organizations received tax credits in exchange for donations to proposed community development initiatives. This has enabled UPMC ISD to give millions of dollars to local community development corporations that undertake housing developments or improvement projects in local communities that are experiencing economic distress.

Land donations and favorable term leases are other giving pathways that healthcare systems can utilize to directly support affordable housing development. As a healthcare system, UPMC makes substantial investments in facilities and land parcels for operations and expansion. Properties without immediate needs can be donated to community developers. For example, UPMC ISD has committed $2.2 million in land donations and favorable long-term leases for affordable housing in two of the city’s most under-resourced and isolated communities. Favorable leases can be used to advance housing programs for those who overrepresent housing insecurity. In 2022, UPMC ISD provided a long-term property lease to the amount of $1 to a local private developer and an aging services provider to create a 52-unit Lesbian, Gay, Bisexual, Transgender, and/or Queer/Questioning (LGBTQ)-friendly senior housing facility near city hospitals, clinics, and community resources. 

Other organizational assets (e.g., equipment and medical or business expertise) can also be donated to community housing initiatives. For example, UPMC ISD committed $7 million in free healthcare services, equipment, and expert consulting, and in partnership with other Pittsburgh-based healthcare systems, local financial institutions, and philanthropic organizations, created a shelter for those experiencing homelessness in Pittsburgh’s downtown community.

## 4. “Partner”

Pennsylvania’s Medicaid transformation stimulated innovation and improvements in UPMC ISD’s existing housing partnerships and has provided new opportunities for the primary prevention of housing insecurity and its associated impact on health outcomes and inequities. With these changes to Medicaid, UPMC ISD could use Medicaid premium dollars for activities such as care coordination to supplement other funding sources, which could sustain and scale housing programs, such as Cultivating Health for Success. While HUD Housing Choice Vouchers or other available subsidies assist with rent or housing costs, Medicaid funds allocated to UPMC ISD can subsidize other program expenses. UPMC is also exploring the impact of value-based payment arrangements on housing program outcomes. Within the current iteration of Cultivating Health for Success, partner housing organizations earn bonuses if they achieve predetermined housing outcomes, such as finding housing within a specified period or securing long-term housing. 

These close partnerships with housing organizations are important for ensuring that those with Medicaid who are eligible for housing assistance can access, apply for, and retain affordable housing. UPMC ISD has enrollment and health information for individuals with Medicaid, and community-based housing partners have familiarity with local communities and existing housing assistance referral networks, experienced and knowledgeable staff who assist with completing housing applications, identifying and moving into new housing, and introducing UPMC ISD care managers to help connect other forms of social assistance and health services. Within programs like CHFS, participants are periodically assessed (e.g., every six months) for improvements in their social and health needs. Individuals and families who desire to and are capable of transitioning from supportive housing into other housing options “graduate” from the program with continued, though appropriately less intensive, support from UPMC ISD and its community housing partners. 

Individuals with low incomes, complex medical needs, functional limitations, or all three frequently experience (1) interruptions in care when transitioning from the inpatient hospital or facility care setting to home or (2) structural barriers that compromise safety and health at home [26]. UPMC ISD supports community and other care-related transitions through intensive care management programs—staffed by nurse case managers, advanced practice providers, and social workers—to ensure, upon their discharge to home, that people have the required services, equipment, and medical care that is critical to their recovery, health, and function [27]. Community Health Workers (CHWs) are also integrated within these transition teams through partnerships with CBOs that train and employ them. CHWs serve as an essential and trusted community resource [28] and perform outreach and check-ins for those who may have a distrust of medical institutions or experience difficulty with direct communication. For those experiencing homelessness at the time of discharge from a medical setting, UPMC ISD financially supports local medical respite programs and community shelters to ensure that safe housing is available at the time of transition of care.

Individuals in community dwellings and with low incomes must often choose between home safety modifications or repairs, other necessities, or medical care. For those with Community Health Choices insurance, UPMC ISD contracts with CBOs who specialize in home adaptations and repairs to prevent the exacerbation of existing medical conditions. For example, these organizations make roof repairs, perform mold remediation, install home equipment to improve safety, or make rooms or entrances more accessible upon the transition from care to home [29,30,31].

UPMC ISD also uses Medicaid funds to finance innovative community partnerships that address upstream housing risk factors. Through a medical–legal partnership with UPMC ISD [32], adults with Medicaid insurance can access free legal services to resolve housing and energy access issues, habitability, and abuse and exploitation that may cause forced displacement. Other partnerships with statewide non-profits help adults with low incomes to obtain cash assistance and additional benefits through Pennsylvania’s social assistance benefit programs (e.g., Supplemental Nutrition Assistance Program (SNAP), Low-Income Home Energy Assistance Program (LIHEAP), and Temporary Assistance for Needy Families (TANF)) to overcome any enrollment associated barriers such as stigma, a lack of information, access, or time. UPMC ISD recently began a similar program with a social startup company that engages people at local laundromats. Program staff use this period of personal downtime at the laundromat to help adults enroll in social assistance programs or schedule appointments with healthcare providers. During the COVID-19 pandemic, UPMC ISD provided funding to local Emergency Rental Assistance Program (ERAP) [33] administrators in six counties to expand their staffing capacity and enhance their outreach capabilities. In just three months in one county alone, these ERAP administrators expedited over 1600 applications.

In addition to community-based programs, UPMC ISD has created innovative hiring programs, such as Pathways to Work, that foster employment and economic mobility for those with Medicaid insurance while simultaneously addressing workforce shortages throughout the IDFS. A specialized team of recruiters assists with resumes and job applications, assesses skills and job fit, directs people to workforce training opportunities, and supports workplace engagement and job retention. Since its launch in 2021, Pathways to Work has assisted over 5000 people who were previously unemployed to find and gain employment within the UPMC healthcare system. UPMC ISD also financially and programmatically supports Freedom House 2.0: a program that recruits, trains, and employs first responders from under-resourced Pittsburgh communities. Freedom House 2.0 is based on an historic Pittsburgh program that sought to create economic opportunities for Black men and women during the 1960 and 1970s that left a lasting legacy on first responder training in the United States [34].

## 5. “Invest”

Healthcare system investment funds can close funding gaps for housing development projects and other social impact initiatives performed in partnership with CBOs, the government, and other healthcare systems [35]. In 2017, UPMC ISD began to make mission-driven housing investments with community development financial institutions (known as CDFIs)—financial lenders that promote and engage in fair and responsible lending with the goal of extending affordable financing to community developers, businesses, and individuals from under-resourced communities [36]—and other financial organizations and non-profits in the local region. Pooling loan funds for dedicated housing investment funds is mutually beneficial for all involved entities. Financial contributors like the UPMC ISD healthcare system bears less financial risk when extending loans to community housing developers and can potentially benefit from long-term returns on investment by creating healthier and more economically stable communities. Mission-driven housing developers receive access to low-interest capital that might be difficult to obtain from traditional lenders and benefit from revenue generated from new or revitalized housing. Communities are improved as abandoned homes are repurposed into affordable homes for low-income individuals and families. 

For example, within the past five years, UPMC ISD has committed $15 million from its insurance reserves to multiple investment initiatives that include: (1) a private loan pool that was conducted in partnership with several prominent Pittsburgh area foundations to preserve 1200 units of affordable housing; (2) a pledge made through a national learning community funded by the Robert Wood Johnson Foundation to accelerate the preservation or production of 1000 housing units over the next 10 years; and (3) contributions to the Pittsburgh Urban Redevelopment Authority’s small landlord fund. The latter is an innovative fund that commits small, low-interest loans to landlords who need to renovate owned properties and qualify for the Housing Choice Voucher program [37]. Landlords commit to a period of affordability for low-income individuals in exchange for favorable loan conditions. 

## 6. Challenges

The healthcare industry’s transition from volume to value has necessitated changes to policy, infrastructure, and healthcare practice [38]. Substantial resources have been invested to redesign information systems, transform care delivery workflows, and modify reimbursement practices and policies [39]. Analogous efforts are needed to achieve and sustain integration across healthcare and social service delivery settings and overcome implementation barriers [40]. In the paragraphs below, we review the most common implementation barriers encountered by UPMC ISD.

### 6.1. Privacy Regulations

To be effective, community-healthcare partnerships require the rapid identification of shared populations and seamless information exchange. Ambiguous and restrictive privacy regulations, although enacted with good intentions, impede information sharing, increase project time and cost requirements, stifle innovation, and place additional burdens on front-line staff and service recipients [41]. Current federal privacy regulations narrowly define case management and care coordination activities—activities that are critical for advancing both treatment and social assistance—and do not explicitly describe circumstances in which healthcare systems can readily share protected health information with CBOs, as they would with other covered entities for the purposes of care delivery [42]. In some areas of the United States, state laws governing privacy for high-risk populations (e.g., those with behavioral health conditions) are more stringent than federal regulations, which can cause delays in care [43]. Furthermore, CBOs often have limited knowledge of privacy requirements or data infrastructure for compliance [44]. Consequently, healthcare systems have been hesitant to make protected health information (PHI) disclosures to CBO partners as they do with other covered entities [45]. Encouragingly, federal and state governments are heeding calls for privacy reform. The Department of Health and Human Services has proposed and passed amendments that clarify disclosures to CBOs, and states with more restrictive behavioral health privacy laws have begun to amend them to achieve parity with federal regulations [46].

### 6.2. Data Quality and Silos

The healthcare industry lacks a standardized and universal approach to assessing social risk factors and social service needs [47]. Standardization is needed so that social investment returns and intervention effects can be consistently measured across organizations, delivery settings, and geographies [48]. In the United States, the Centers for Medicare and Medicaid Services (CMS) and some state Medicaid programs are gradually introducing new screening measures for social risk factors within value-based payment programs for healthcare systems and healthcare providers [49,50].

Screening data alone, however, are often insufficient for coordinating care across service settings. Healthcare providers are often unaware of the social services that an individual is already receiving or whether a patient follows through with social service referrals [51]. Likewise, CBOs and public health agencies are generally unaware of a service user’s medical history and treatment needs. Broader and mutually beneficial information exchange between healthcare systems and county or state health and human services departments—who maintain applicable program registries for social assistance programs—could potentially address this issue and provide healthcare systems and their community partners with more actionable data. UPMC ISD has successfully established such a relationship with one local county human services department. To accomplish this, the two entities entered into a data use agreement that ensured privacy controls and infrastructure that have been in place for securely transmitting and storing data, which defines how each party may use the data that they receive. A cross-organizational workgroup was also established to develop and standardize processes, documentation, and variable definitions to improve the utility of integrated data and ease data-sharing processes. This workgroup meets regularly to identify and review progress for data use cases with the potential to improve service quality. Social service data that UPMC ISD receive have been used to improve care coordination for individuals with intellectual disabilities/autism, optimize the work of CHWs by directing them to individuals with immediate social service needs, and improve screening, preventive service use, and access to community services and support for individuals receiving care from UPMC’s women’s health providers. 

### 6.3. Funding

Payment reforms like those within the Pennsylvania Medicaid program have enabled healthcare systems to reallocate premiums from medical services to community programs. Investment opportunities and their impact, however, are constrained by the number of premium dollars that can be reallocated away from medical services. Additional financial resources are needed as healthcare systems become more accountable for addressing and supporting health-related social needs. The current risk adjustment formulas used by CMS and state Medicaid programs rely on medical service utilization and demographic characteristics to calculate payment amounts; however, these formulas do not fully account for social risk factors or anticipated social service needs [52]. Without accurate risk adjustment in the current and proposed value-based reimbursement frameworks, healthcare systems could be underfunded or unfairly penalized [53].

In part, these funding shortfalls could be addressed through braided funding. Braided funding leverages multiple private and public funding sources to finance housing programs and other social impact initiatives [54]. As described earlier, UPMC ISD applies this funding approach to some housing programs. However, the success of this strategy depends on the availability of housing vouchers or other subsidies. The quantities of housing vouchers are often limited with the current levels of government funding [55], and voucher shortages contribute to housing delays and threaten program sustainability. To better support healthcare systems and incentivize investment, housing authorities could prioritize a planned quantity of housing vouchers for healthcare systems that demonstrate needs within well-designed programs. 

## 7. Conclusions

Healthcare in the United States is entering a new era of transformation and reform that is more attentive to health-related social needs and health inequities. With this shift in the paradigm, healthcare systems are poised to help fill gaps in public health infrastructure and address housing insecurity and its associated impact on health outcomes and inequities. Action beyond the clinical setting that could make substantial investments in the structural determinants of health and innovative community-based interventions with high primary prevention potential is needed to positively impact associated health outcomes and inequities. 

The novel framework proposed by UPMC ISD, “Give, Partner, Invest,” and their related implementation examples presented in this manuscript describe methods for healthcare systems to integrate primary housing interventions and investments within their healthcare operations to address unmet housing needs and their associated impact on public health and inequities. While this framework was developed within the context of the United States healthcare system, given the paucity of the literature on this type of public–private approach to addressing housing insecurities, we hope these findings can serve as a tool for healthcare systems in the advanced economies of the world to inform housing policy reforms and potentially catalyze change. Further, we hope that the publication of our experiences could encourage other healthcare systems and stakeholders to discuss their investments, interventions, and outcomes to improve solutions for those affected by housing insecurity and homelessness and to strengthen the health and well-being of our communities. As UPMC ISD’s housing and community investments grow, we look forward to sharing the successes and challenges of our housing initiatives, innovative partnerships, and their resulting impact on public health and inequities in our communities.
